# Multi-Level Communication of Human Retinal Pigment Epithelial Cells via Tunneling Nanotubes

**DOI:** 10.1371/journal.pone.0033195

**Published:** 2012-03-22

**Authors:** Dierk Wittig, Xiang Wang, Cindy Walter, Hans-Hermann Gerdes, Richard H. W. Funk, Cora Roehlecke

**Affiliations:** 1 Institute of Anatomy, TU Dresden, Dresden, Germany; 2 Department of Biomedicine, University of Bergen, Bergen, Norway; 3 CRTD/DFG-Center for Regenerative Therapies Dresden – Cluster of Excellence, Biotechnology Center, Dresden, Germany; University of Colorado, Boulder, United States of America

## Abstract

**Background:**

Tunneling nanotubes (TNTs) may offer a very specific and effective way of intercellular communication. Here we investigated TNTs in the human retinal pigment epithelial (RPE) cell line ARPE-19. Morphology of TNTs was examined by immunostaining and scanning electron microscopy. To determine the function of TNTs between cells, we studied the TNT-dependent intercellular communication at different levels including electrical and calcium signalling, small molecular diffusion as well as mitochondrial re-localization. Further, intercellular organelles transfer was assayed by FACS analysis.

**Methodology and Principal Findings:**

Microscopy showed that cultured ARPE-19 cells are frequently connected by TNTs, which are not attached to the substratum. The TNTs were straight connections between cells, had a typical diameter of 50 to 300 nm and a length of up to 120 µm. We observed *de novo* formation of TNTs by diverging from migrating cells after a short time of interaction. Scanning electron microscopy confirmed characteristic features of TNTs. Fluorescence microscopy revealed that TNTs between ARPE-19 cells contain F-actin but no microtubules. Depolymerisation of F-actin, induced by addition of latrunculin-B, led to disappearance of TNTs. Importantly, these TNTs could function as channels for the diffusion of small molecules such as Lucifer Yellow, but not for large molecules like Dextran Red. Further, organelle exchange between cells via TNTs was observed by microscopy. Using Ca^2+^ imaging we show the intercellular transmission of calcium signals through TNTs. Mechanical stimulation led to membrane depolarisation, which expand through TNT connections between ARPE-19 cells. We further demonstrate that TNTs can mediate electrical coupling between distant cells. Immunolabelling for Cx43 showed that this gap junction protein is interposed at one end of 44% of TNTs between ARPE-19 cells.

**Conclusions and Significance:**

Our observations indicate that human RPE cell line ARPE-19 cells communicate by tunneling nanotubes and can support different types of intercellular traffic.

## Introduction

The retinal pigment epithelium (RPE) forms the outer blood-retinal barrier by separating the outer retina from the choroid capillary bed. It is an active barrier that supports the neuronal retina in many ways. RPE supplies trophic factors for the retina, phagocytise disc membranes that are shed daily from the photoreceptors, recycles retinoids to sustain the visual cycle, and regulates the composition and volume of the subretinal space.

Little is known regarding the intercellular signal transduction pathways between RPE cells, in spite of their important physiological functions. Electrophysiological studies have shown that all retinal cells communicate with their neighbours via gap junctions [1], [2], [3], [4]. Cx43-mediated gap-junctional intercellular communication participates in the regulation of retinal organogenesis [5], [6] and regeneration [7]. Some studies have reported that cell death signals can be transmitted through the aqueous pores of gap junctions to adversely affect their neighbours [8], [9], [10], [11], [12], this is called “bystander effect” [13]. This suggests that RPE cells sustain very active intercellular communication under physiological conditions.

In 2004, a new type of cell-cell communication between animal cells, based on the formation of thin intercellular membrane channels, was reported in the neuron-like pheochromocytoma cell line PC12 [14]. These highly sensitive nanotubular structures were termed tunneling nanotubes (TNT), connecting individual cells and facilitating selective long-range cell-cell communication. Later, TNT formation has been observed in immune cells, including B, T and NK cells, neutrophils and monocytes, as well as in neurons, glia, cultured prostate cancer cells and cardiac myocytes [15], [16].

So far, proposed functions of TNTs are long-distance exchange of cellular compounds, ranging from small endosomes up to large organelles, cytoplasmic molecules, calcium signals, vesicles and thereby coordination of signalling between TNT connected cells [15]. Furthermore, a growing number of reports implicate TNTs as pathways for pathogens, such as bacteria, viruses and prions indicating that TNTs might also play a role in diseases [16]. Thus, TNTs vary in diameter, length, architecture and functions in diverse cell types [17], [18], [19].

In the present study we provide first evidences for TNTs in RPE cells. We investigated TNT structure and functions using the retinal pigment epithelial cell line ARPE-19, a nontransformed adult human RPE cell line, which retains many of the morphological features of RPE [20].

## Results

### Analysis of characteristics of TNTs

**Figure 1:**
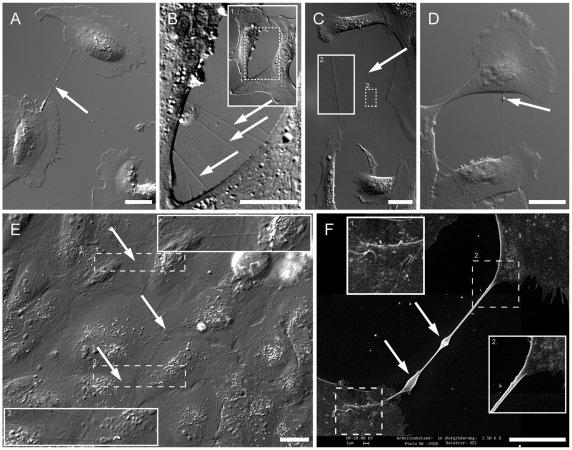
The presence of membrane nanotubes in live ARPE-19 cells. (A) Two ARPE-19 cells are directly connected with a straight membrane tube (arrow). Scale bar, 20 µm. (B) High magnification of 3 separate straight membrane tubes (arrows) connecting two ARPE-19 cells. Inset 1 is an overview of the two connected cells. Scale bar, 20 µm. (C) A long nanotube (120 µm, arrow) connects ARPE-19 cells. The inset 2 shows the tube in a higher magnification. Scale bar, 20 µm. (D) A bulge (arrow) locates at the upper end of the tube. In time lapse videos the bulges moved along the tube from one cell to the other. Scale bar, 20 µm. (E) Thin membrane tubes (arrow) form and locate above the confluent layer of ARPE-19 cells after 48 h cultivation. The insets 1 and 2 show the tube in a higher magnification. Scale bar, 20 µm. (F) Scanning electron microscopy (SEM) shows the ultrastructure of a TNT between two ARPE-19 cells. Enlarged views (inset 1 and 2) show smooth membrane between the nanotube and the plasma membrane of the two connected cells. The TNT forms a straight connection between two cells and has a typical diameter of 250 nm. Arrows mark two focal thickenings of 1 µm indicating a possible transport of organelles or vesicles through the nanotube. The picture was taken with 20 kV. Scale bar, 10 µm.

To identify TNTs in the human RPE cell line ARPE-19, we used differential interference contrast (DIC) microscopy to avoid phototoxic damage to these fragile structures. Strinkingly, sub-confluent ARPE-19 cells were frequently connected by TNTs (Figure 1A, B, C, D). In the majority of cases one straight TNT linked two cells with each other, but occasionally up to three or four distinct TNTs were found (Figure 1B, arrows). The frequency of nanotubes connected cells was 14.3% in ARPE-19 cell culture (n = 1200). The mean length of TNTs was 43.6±18.1 µm. The longest TNT was over 120 µm (Figure 1C). Interestingly, the ends of TNTs are often close to cell nuclei as shown in Figure 1B, C and D. We observed bulges along the lengths of some nanotubes (Figure 1D). Focal bulges or vesicle travelling along nanotubes are previously suggested means of cell-cell exchange.

TNTs maintained straight configuration during the cell migration (Movie S1). We demonstrated by DIC that TNTs form on the upper side of confluent ARPE-19 cells (Figure 1E, arrows, insets). TNTs frequently connected two cells and were freely moving in the medium without direct contact to cells below or to the surface of the culture dish, which is a major attribute distinguishing TNTs from filopodia. Scanning electron microscopy confirmed a characteristic feature of TNTs: a typical diameter of 50 to 300 nm (Figure 1F, insets). In addition, the size of bulges along the TNT were thicker than the diameter of TNTs (Figure 1F).

### Formation of TNTs

**Figure 2:**
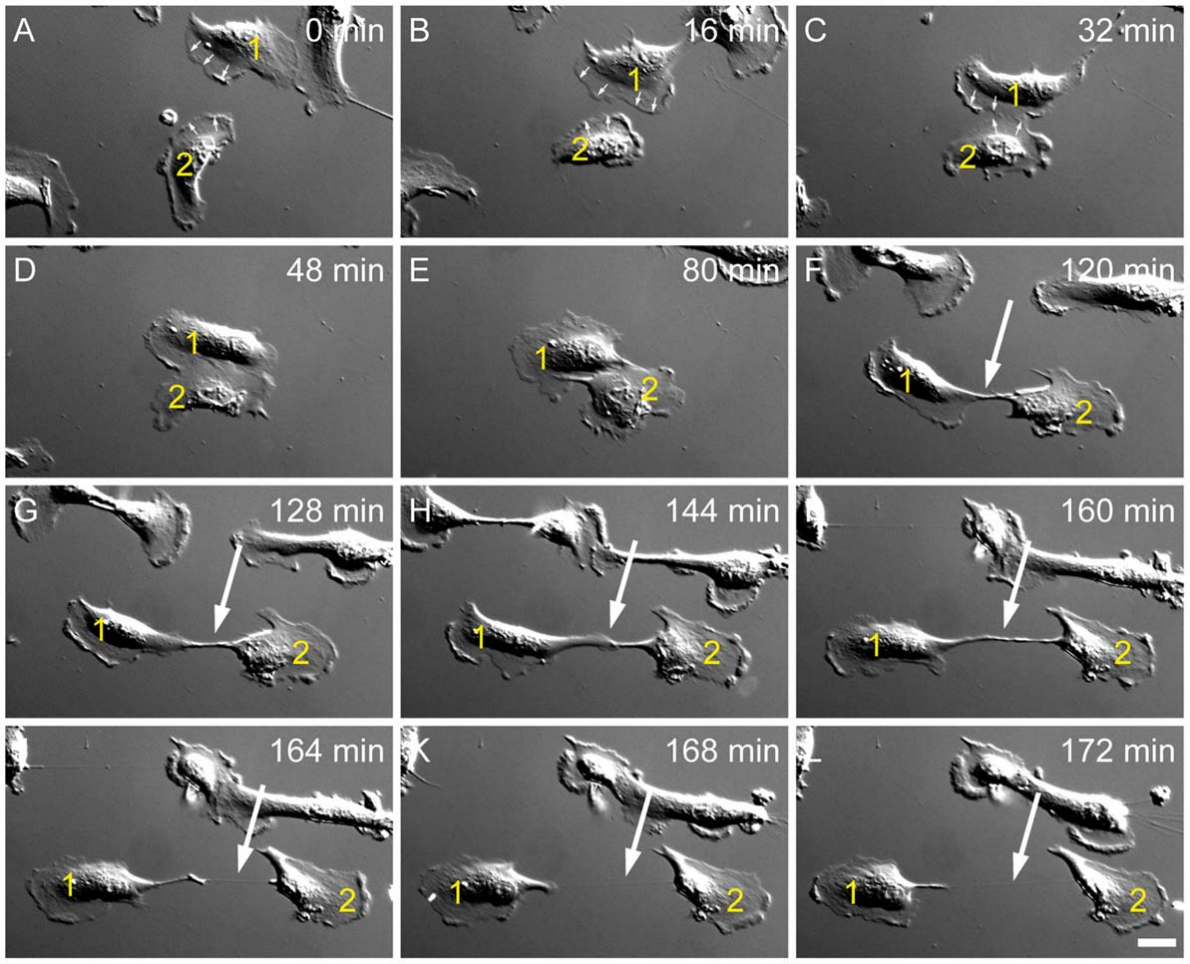
The formation of nanotube between migrating ARPE-19 cells. (A-C) Two separated cells (cell 1 and 2) migrate towards each other and making contact after 32 min. Note the leading front (small arrows) and the trailing end with the nucleus. (D-F) Cell 1 and Cell 2 keep the contact over 60 min and start to diverge in (F). (G-I) Between the two cells a connection (arrow) still remains and becomes longer with the distance of the separating cells. (J-L) The connection between the cells becomes smaller and longer and has now a hair like shape (arrows in J and H). Finally, the connection form a tunnelling nanotube with a size of 70 µm (arrow in L). Scale bar, 20 µm.

To investigate the formation of TNTs, we monitored ARPE-19 cells for more than 24 hours by time lapse DIC microscopy. As shown in Figure 2, TNTs formed after contact of migrating ARPE-19 cells. The cells initially have contact (Figure 2B, 2C, 2D), followed by a dislodgment and a visible cell-cell connection that elongates as the cells diverge (Figure 2F), which subsequently result in a TNT (Figure 2L). The formation of a membrane nanotube between ARPE-19 cells requires the presence of a close-by target cell, since no membrane nanotubes were observed on individual cells at distances larger than 100 µm from their neighbours. This was confirmed in 18 experiments with 49 TNT formations. We observed a disassembly of nanotubes severed after a major dislodgment of migrating cells. Lastly, both final ends curled up and were withdrawn. The lifetime of these TNTs ranged from minutes up to an hour. Surprisingly we did not observe any formation by outgrowth of protrusions like previously reported for other cell types e.g. PC12 cells [14]. This fact reveals that TNTs between ARPE-19 cells are generated upon dislodgment of cells after a short time of interaction.

### TNTs contain F-actin but no microtubules

**Figure 3:**
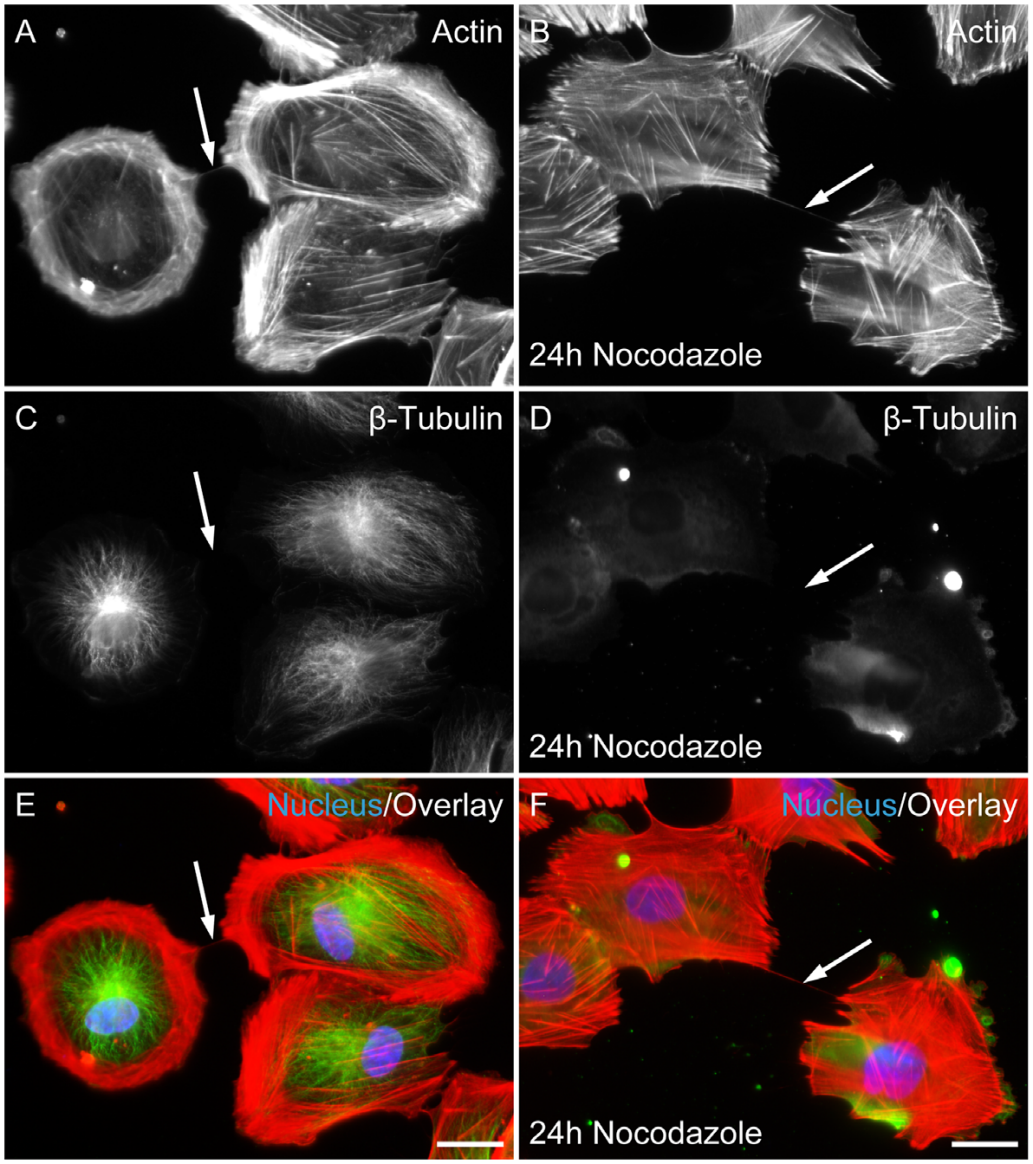
Membrane nanotubes of ARPE -19 cells contained F-actin but not microtubules. Fluorescence image of ARPE-19 cells stained with phalloidin-TRITC for actin (A, B) anti-ß-tubulin (C, D) and nucleus (DAPI). Images represent the cytoskeleton in control (no treatment A, C, E) and after treatment with 15 µM nocodazole for 24 h (B, D, F). (A, B) Fluorescence image of F-actin. Actin fibres were visible in the cells and clearly seen is a straight connection, representing the TNT structure, between the cells (arrows). (C, D) Fluorescence image of ARPE-19 cells stained with mAb against β-tubulin revealed the lack of β-tubulin in the TNTs (arrows). (E, F) Merged pictures with actin (red), ß-tubulin (green) and nucleus (blue). Arrows mark TNTs. Scale bar, 20 µm.

It has been previously shown that nanotubular connections may be heterogeneous in their cytoskeletal structure [17], [21]. Immunostaining showed that TNTs contained only F-actin but no β-tubulin in ARPE-19 cells (Figure 3A, C, E, n = 43). To test this finding, cells were incubated with actin-depolymerising drug latrunculin B or microtubules-depolymerising drug nocodazole. Addition of latrunculin B stopped the *de novo* formation of all TNT-like structures and resulted in complete disappearance (data not shown). Thus, F-actin is essential for maintaining TNT integrity between ARPE-19 cells. In contrast, TNTs were readily detected in the presence of nocodazole (Figure 3B, D, F) and a quantitative analysis revealed that this drug did not considerably change the number of TNTs. In control cells, 17.2 TNTs per 100 cells (n = 418) were counted, whereas 16.1 TNTs per 100 cells (n = 310) were identified in the presence of nocodazole. Thus, the formation of TNTs in ARPE cells is independent of microtubules.

### Transmission of calcium signals through TNT-like structures

**Figure 4:**
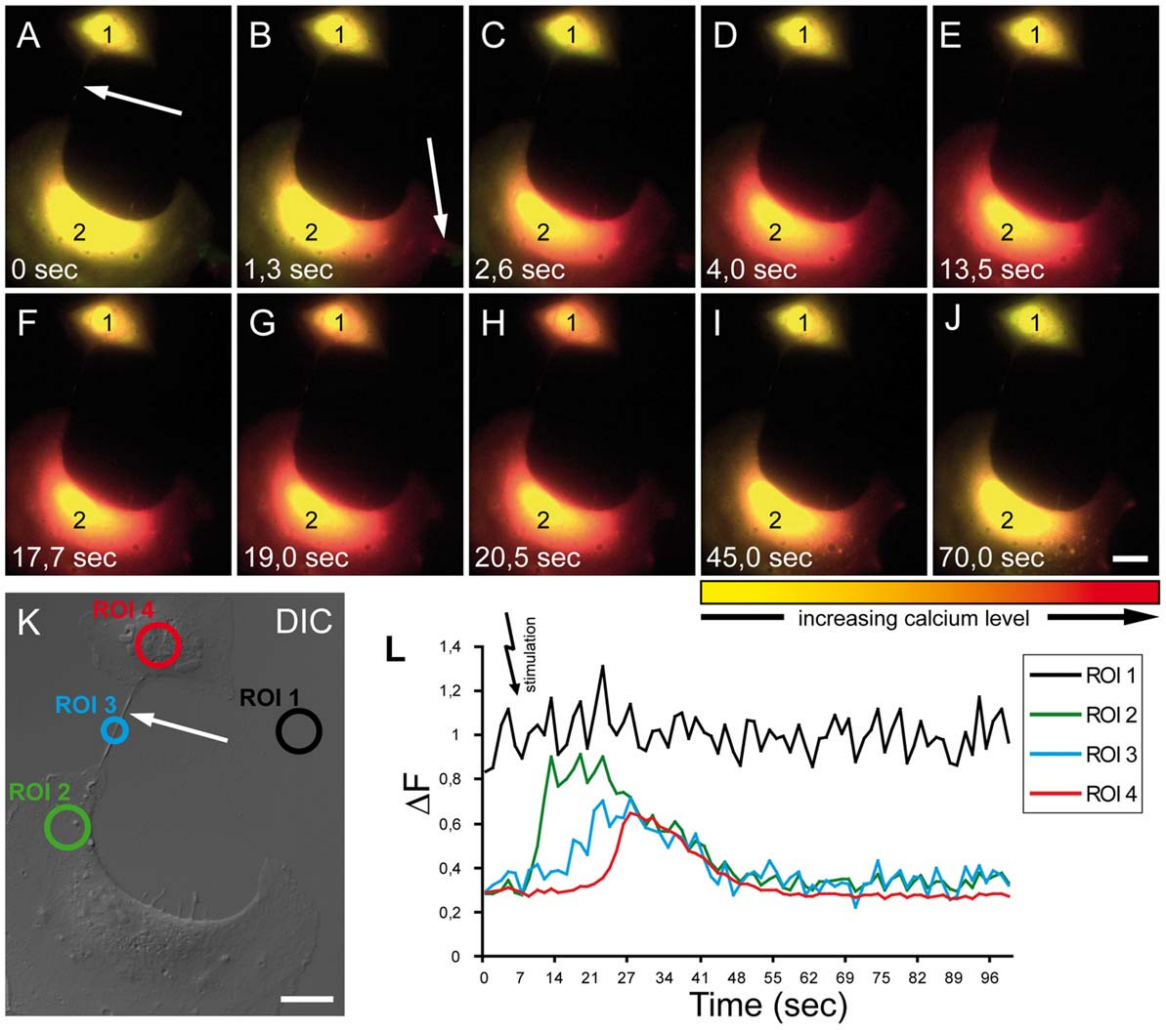
Intercellular Ca^2+^ flux between TNT connected ARPE-19 cells. (A-J) After mechanical stimulation of cell 2 (arrow in B), the intracellular Ca^2+^ level increases in cell 2 (B-D). After 13.5 sec of delay, a transmission of Ca^2+^ via a TNT (arrow in A and K) reached the connected cell 1 (E-F). The high level of Ca^2+^ is now clearly seen in cell 1 (G-H) After 45 sec the Ca^2+^ level in both cells recover close to the normal level (I-J) Colour bar indicates relative level of Ca^2+^ measured by the Ca^2+^ indicator Fura-2 AM. Scale bar, 20 µm. (K) DIC image shows the two cells connected via a TNT (arrow). Concentration of Ca^2+^ was measured in the regions of interest (ROIs, circles): stimulated cell (green, ROI 2), TNT (blue, ROI 3), TNT-connected cell (red, ROI 4) and background (black, ROI 1). Scale bar, 20 µm. (L) Quantification of the relative Ca^2+^ concentration (ΔF) within the ROIs in (K) The background level of Ca^2+^ is subtracted in ROIs 2, 3 and 4. The Ca^2+^ level in cell 2 increase directly after manipulation and reaches a peak after 7 sec. At this time point the Ca^2+^ concentration of the TNT (ROI 3) starts to increase and reaches a peak 14 sec after manipulation. After this the Ca^2+^ level in cell 1 increase to a maximum 20 sec after manipulation. Both cells recover to normal level of Ca^2+^ (27–100 sec). A typical result was shown from 5 independent experiments.

In some cell types nanotubular connections have the capacity of transferring calcium signals among connected cells [19], [22], [23], [24]. Here we evaluated the intercellular transfer of calcium signal via nanotubes in ARPE-19 cells. Intracellular calcium fluxes were induced by physical stimulation with a micromanipulator. Cells were loaded by a fluorescent calcium indicator dye and using DIC microscopy we selected cells connected by a TNT (Figure 4K). Fluorescence images were taken every 1.3 seconds (Figure 4A, B, C, D, E, F, G, H, I, J, Movie S2). The calcium signal moved from the touched edge to the other end of the manipulated cell (Figure 4B, C, D). After a short delay, an Ca^2+^ level increase was observed in the TNT. Depending on the length of the TNT structure, the calcium signal transmitted to the second cell was temporally delayed resulting in an increased intracellular Ca^2+^ level (Figure 4G). Surrounding cells, which were not connected to the stimulated cell, were not affected. After a short time, connected and stimulated cells repolarised to Ca^2+^ base level (Figure 4H, I, J). Ca^2+^ levels were analyzed in 3 different regions of interest (ROI) (Figure 4K, L). The Ca^2+^ increase in the receiving cell was lower compared to the stimulated cell. Interestingly repolarisation occurred simultaneously in TNT, receiving and stimulated cell.

### Intercellular transfer of small molecules via TNTs

**Figure 5:**
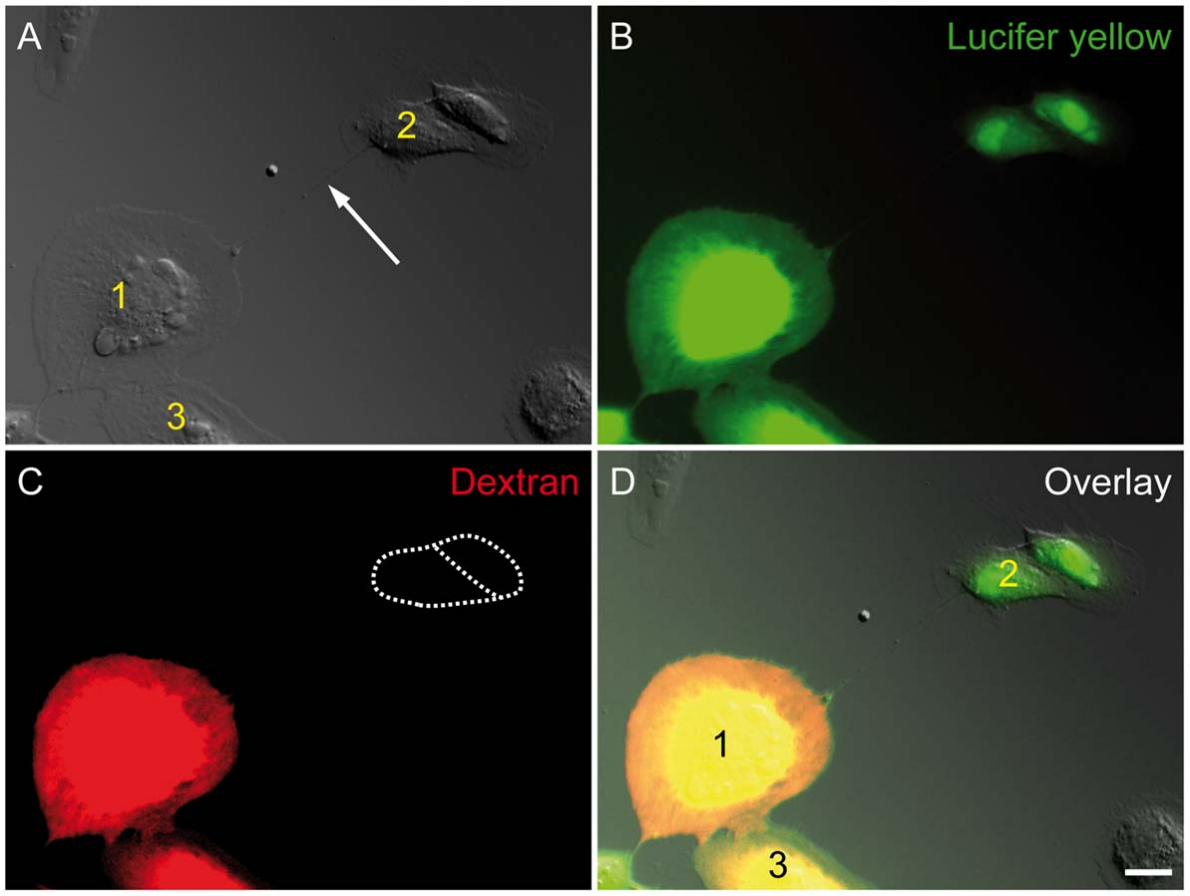
Intercellular transfer of small molecules via a TNT of ARPE-19 cells. (A) The DIC image show a membrane tube (arrow) and two connected ARPE-19 cells (1, 2). (B-C) Cell 1 and Cell 3 were injected with a mixture of Lucifer yellow (green, B) and Texas red Dextran 10,000 (red, C). The fluorescence images taken 10 min after injection show that only Lucifer yellow was transmitted to cell 2 (green, B), while Texas red Dextran was not detected in cell 2 (dashed area in C). A typical result was shown from 5 independent experiments. Scale bar, 20 µm. (D) The overlay of DIC, the Lucifer yellow-positive cells and the Texas red Dextran positive cells, and the membrane tube (arrow in A).

To test whether molecules transferred by TNTs are subject to any size restrictions, we used a small molecule Lucifer Yellow and Texas Red Dextran as a larger molecule. When two cells (Figure 5, cell 1 and cell 3) were microinjected with a mixture of Lucifer Yellow and Texas Red Dextran, Lucifer Yellow was transmitted to cell 2 via TNT following microinjection in cell 1 (Figure 5, cell1 and cell 2), but Texas Red Dextran stayed within the source cells (Figure 5, cell 1 and cell 3). Lucifer Yellow was faintly detectable within the connecting TNT, possibly due to the small lumen. The different diffusion levels of Lucifer Yellow and Texas Red Dextran in TNTs indicated that TNTs in ARPE-19 cells might function as channels for small molecules, but not large molecules.

### Mitochondria are detected within membrane nanotubes

**Figure 6:**
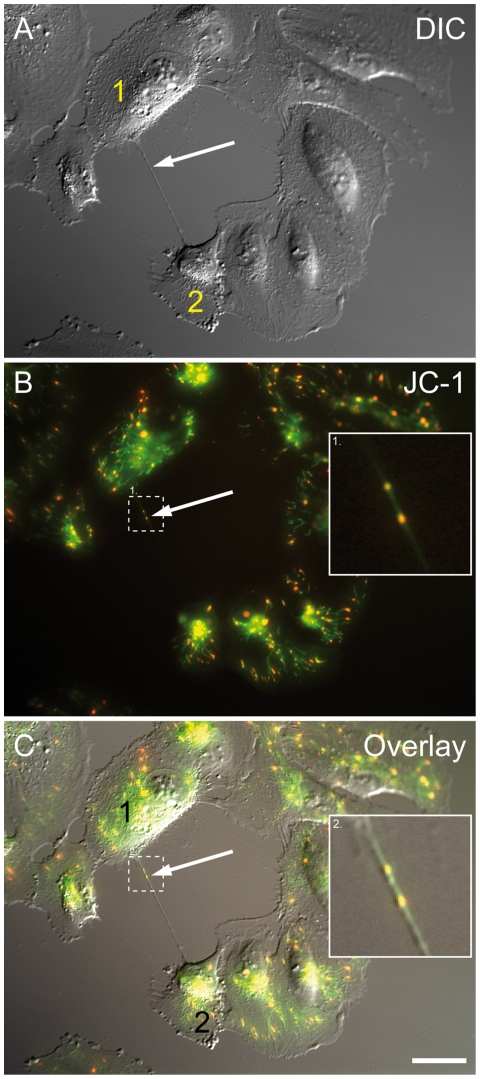
ARPE-19 cells connected by a nanotube containing mitochondria . (A) The bright field image shows two ARPE-19 cells connected by a membrane nanotube (arrow). (B) The corresponding fluorescence image of (A) shows JC-1 labelled mitochondria of cells (arrow). (C) The overlay of (A) and (B) shows the co-localization of nanotube with fluorescent labelled mitochondria (enlarged box). Scale bar, 20 µm.

The observations of bulges along TNTs suggested the presence of organelles in TNTs. We therefore addressed the possibility of mitochondria transfer within nanotubes of ARPE-19 cells. By using the specific mitochondrial dye JC-1, we indeed observed fluorescently labelled mitochondria inside TNTs of living cells (Figure 6). This suggests that ARPE-19 cells have the capacity of organelle transfer via TNTs.

### Endocytic organelle transfer between ARPE-19 cells

**Figure 7:**
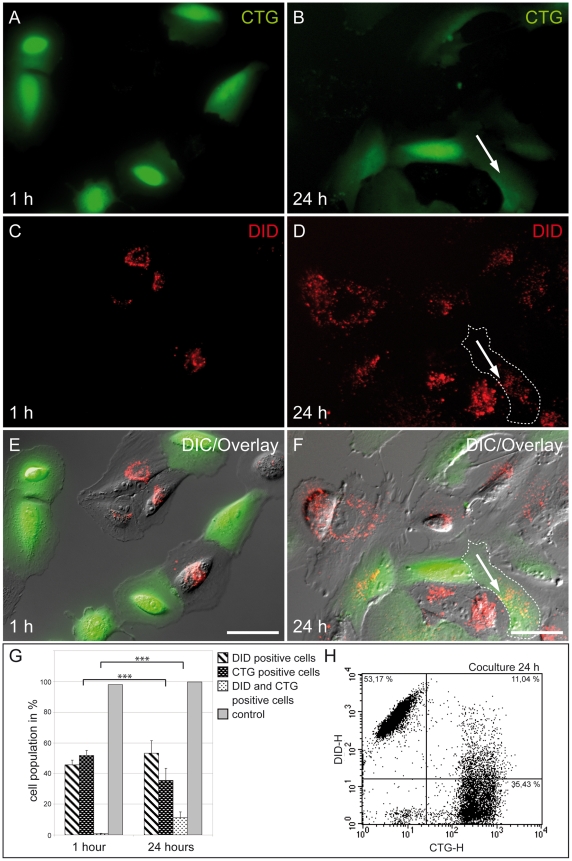
Transfer of endocytic organelles between ARPE-19 cells. (A-F) Mixed populations of CTG (green) and DID (red) labelled ARPE-19 cells were cocultured for up to 24 hours and analyzed by fluorescence microscopy. As acceptor population, cells were labelled with CTG (A-B). As an organelle donor population, cells were labelled with DiD leading to red fluorescently labelled endocytic organelles (C-D). After populations were mixed, images were obtained 1 hour (A, C, E) and 24 hours (B, D, F) after seeding. The arrows show identical cells in panels B, D, F, respectively. Double-positive cells are indicative of organelle transfer (arrow in F). Scale bar, 20 μm. (G-H) FACS analyses revealed a significant (p < 0.001, t-test) transfer of 11.04% labelled endocytic organelles after 24 hours cultivation in comparison to 1 hour. A representative dot plot from the flow cytometry analysis is shown in (H).

To assay intercellular organelle transfer in ARPE-19 cells, we used a coculturing system consisting of organelle donor and organelle acceptor cell populations. As an organelle donor population, cells were labelled with red fluorescent DiD leading to red labelled endocytic organelles. Organelle acceptor populations were labelled with green fluorescent CellTracker (CTG) which is not transferable between cells. Images of the cocultures were taken one hour after cells attached and 24 h after seeding (Figure 7). After one hour CTG labelled cells and DiD labelled cells could be clearly distinguished from each other (Figure 7E). After 24 hours of incubation DiD positive organelles were occasionally found in acceptor cells (Figure 7F, arrow). Double-positive cells are indicative of organelle transfer. Flow cytometry analyses (FACS) revealed a significant (p<0.001, t-test) transfer of 11.04% of endocytic organelles after 24 hours cultivation in comparison to one hour (Figure 7G, H). In six independent experiments we demonstrated that 23.94±7.99% of all CTG positive acceptor cells received endocytic organelles from donor cells.

**Figure 8:**
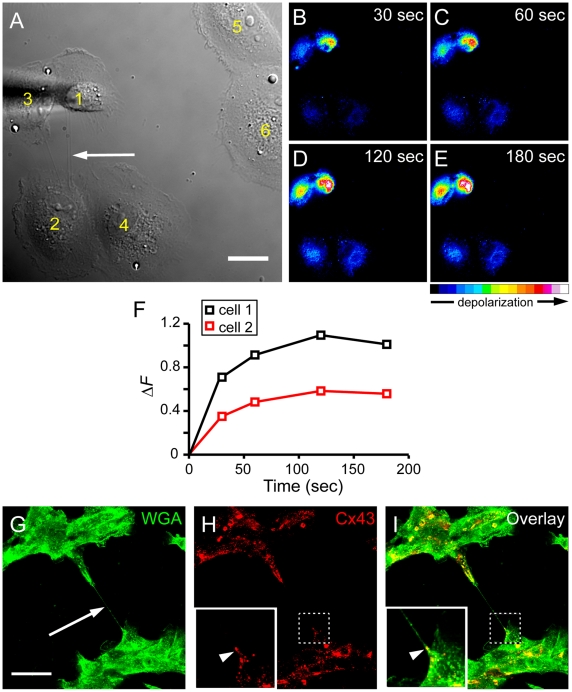
Depolarisation signals spread between TNT-connected ARPE-19 cells. (A) The DIC image shows the mechanically stimulated cell (cell 1), TNT-connected cell (cell 2), TNTs (arrow) and control cells (cell 5 and 6). Scale bar, 20 µm. (B-E) The pseudo-coloured intensity images, generated by subtraction of the image before stimulation, show DiBAC4(3) fluorescence increase of cells in (A) at indicated times after mechanical stimulation. Note that the close associated cells pairs (cell 1 and 3, cell 2 and 4) are also electrically coupled. Colour bar indicates relative level of depolarisation. (F) Quantification of the relative membrane potential changes ( ΔF) of the stimulated cell (cell 1) and the TNT-connected cell (cell 2) as shown in (B-E). (G-I) The presence of Cx43 on TNTs in ARPE-19 cells. Cells were fluorescently labelled using WGA (green; G, I) and anti-Cx43 (red; H, I) and imaged by confocal microscopy. The enlarged images show distinct signals of Cx43 immunolabelling (arrowheads) at one end of a TNT (arrow). Scale bar, 50 µm.

### Mechanical stimulation-induced depolarisation spreads through TNT connections

It has been shown in several cell types that electrical signals are transferred through TNTs [25]. To study whether ARPE-19 cells are also electrically coupled by TNT connections, we depolarised one cell of TNT-connected cell pairs by mechanical stimulation (Figure 8A). Depolarisation of both cells was measured by the membrane potential sensitive dye DiBAC_4_(3). The fluorescence of the stimulated cell and the TNT-connected cell increased after mechanical stimulation (Figure 8B, C, D, E), while control cells (lacking physical connections to the stimulated cell pair) did not display an increase in fluorescence. This suggests a TNT-dependent electrical coupling in ARPE-19 cells. The amplitude of depolarisation in the recipient cell was always lower than that of the stimulated cell (Figure 8F), comparable to previous observations in NRK cells [25]. We detected three electrical coupled cell pairs among 10 tested TNT-connected cell pairs. Further, gap junctions were present on 44% of all observed TNTs (n = 25) illustrated by immunolabelling of punctate Cx43 signals on one end of the TNT (Figure 8G, H, I). This indicates that TNT-dependent electrical coupling in ARPE-19 cells may follow the same mechanism as in NRK cells [25].

## Discussion

TNTs are thought to represent a general and important communication route between cells [17]. They allow cells to functionally interact with target cells over long distances. Numerous studies reported on TNT-like nanotubes connecting cell types, including human B cells, natural killer cells and macrophages, rat astrocytes, human dendritic cells and THP-1 monocytes, neonatal rat cardiomyocytes and human progenitor cells, and Jurkat T-cells [15], [18], [19], [21], [24], [26], [27], [28], [29]. Here, we report characteristics of membrane nanotubes formed by the human RPE cell line ARPE-19.

TNTs of ARPE-19 cells were suspended in the medium between connected cells. In subconfluent cultures ARPE-19 cells migrated freely, searching for counterparts to bind or communicate. Our observations suggest that TNT formation might be an efficient way of intercellular communication. The proof of TNTs in a dense cellular environment of confluent ARPE-19 cells suggests similar long distance contacts of RPE *in vivo*. *De novo* formation of TNTs can occur by different mechanisms that vary with cell type. Two distinct mechanisms of *de novo* formation of TNTs were reported so far, i.e. by the directed outgrowth of a filopodium-like protrusion toward a neighbouring cell or by dislodging of attached cells after interaction with another cell [15], [18]. Live cell imaging revealed that all observed nanotubes between ARPE-19 cells were formed as cells separated after an initial close contact.

In ARPE-19 cells nanotubes consistently contained and structurally depended on F-actin. Microtubules were not detected in nanotubes and the number of TNTs was not changed in the presence of nocodazole. This is consistent with previous studies showing that most cell types form TNTs only containing F-actin, and very few of them form heterogeneous TNTs with both F-actin and microtubules [17].

Here we demonstrated intercellular transport of small molecules via TNTs in ARPE-19 cells. The small molecule marker Lucifer Yellow moved between ARPE-19 cells, and the slightly larger Texas Red Dextran remained within the cell, that was microinjected. These results indicate that small molecules in the cytosol might be transferred from cell to cell by diffusion via TNTs. Considering the presence of Cx43 gap junctions at one end of TNTs, the limitation for the free diffusion of big molecules might depend on the exclusion limit of these gap junctions.

As previously published in other cell type mitochondria can move within TNTs between cells [21], [27], [30]. It has recently been shown in cells with dysfunctional mitochondria, that aerobic respiration can be rescued by transfer of mitochondria or mitochondrial DNA from undamaged cells [31]. Thus, one mechanism by which the reported exchange of mitochondria could occur is via membrane nanotubes. In this study, we observed mitochondria within TNTs suggesting that mitochondria can access nanotubes of ARPE-19 cells. This may be an actin-dependent and energy-driven transport [32], but not through gap junctions within TNTs. Several mechanisms have been proposed to explain how organelles cross the membrane interface between TNT and connected cell, including transient membrane fusion model, multi-vesicular body fusion model and phagocytosis model [33]. In agreement with previous studies [32], we also observed an endocytic organelle transfer in ARPE-19 cells.

In this study we provide strong evidence for TNT-dependent intercellular calcium signalling through TNTs in ARPE-19 cells. Thus, transmission of calcium signals in ARPE-19 cells is not limited to intimate contact [22], [24]. Himpens et al. [34] demonstrated intra- and intercellular calcium signalling in retinal pigment epithelial cells during mechanical stimulation, which was mediated by stretch-sensitive cation channels followed by intracellular calcium release. Calcium signalling between TNT-connected cells may occur by different mechanisms including Ca^2+^ diffusion via TNTs [22], [24], by active propagation of calcium signals by inositol triphosphate (IP_3_) receptors within TNTs [23] or by temporary increase in intracellular Ca^2+^ concentration through the opening of calcium channels in the plasma membrane [25]. We observed that calcium activation in the stimulated cell consistently resulted in Ca^2+^ level elevation in the TNT-connected cell. Unlike Wang et al. [25], who found Ca^2+^ elevation in the TNT-connected cell already after 8 seconds, we found Ca^2+^ elevation after approximately 15 sec at the earliest. In addition, we observed that there was a transient Ca^2+^ peak inside TNTs (Figure 4L). These findings suggest that Ca^2+^ level elevation in the connected cell is more likely caused by Ca^2+^ diffusion through the connecting TNT than by Ca^2+^ elevation via calcium influx through low voltage-gated Ca^2+^ channels of TNT connected cells after stimulation.

We show indications that the human RPE cell line ARPE-19 has TNTs for the exchange of molecular information between cells. This demonstrates that membrane nanotubes between ARPE-19 cells can support different types of intercellular traffic. It may provide a new way of communication between RPE cells or between RPE cells and photoreceptor cells. Compared to other modes of transmission it includes fast electrical communication via TNTs. It also facilitate more specific calcium flow between cells compared to other long distance calcium signalling such as the ATP-purinergic receptor system [35]. Moreover, the intercellular transfer of mitochondria through TNTs suggests a higher level of cell-cell interaction. Thus, the transfer of signals by TNTs and the subsequent activation of biochemical signals may provide a unique mechanism for long-distance cellular signalling in retinal cells.

It is currently unknown to what extent nanotubes exist and which function they have *in vivo*. To date most data are from studies *in vitro*. However, recently it was reported that nanotubes occur between dendritic cells in the mouse cornea, providing the first evidence for their existence *in vivo* [36]. It becomes increasingly apparent that nanotubes fulfil important functions in physiological processes in the organization of multicellular organisms. Some of the signalling processes previously thought being mediated by diffusion may instead result by direct interaction of cellular extensions. For example, cells in the imaginal disc of *Drosophila* can form long actin-rich protrusions called cytonemes, suggested to be important in signalling transduction between cells [37], [38]. Thus, cytonemes are thought to function in long-range signalling between cells during developmental processes.

In the retina, TNTs could mainly serve as tubes for conducting signals or cell compartments between RPE cells or between RPE cells and photoreceptor cells. For example, it is known that RPE cells recycle DHA (docosahexaenoic acid) for biogenesis of disc membranes from phagocytised disc membranes back to the inner segment of the photoreceptor cell [39], [40]. The molecular details of this transport from the RPE cells through the inter-photoreceptor matrix to the inner segment are not yet understood. Hence, one speculative role for retinal nanotubes could be DHA transport.

Our data indicate important roles of TNTs for intercellular communication by electrical coupling, calcium signalling and exchange of cytosolic material in ARPE19 cells. The fact, that to date these structures could not be traced between pigment retina cells *in vivo* may be due to their fragility and small dimensions and the lack of specific makers. Therefore, detection of TNTs *in vivo* and deciphering their function will be of central importance of future investigations.

## Materials and Methods

### Cell culture

ARPE-19 cells (ATCC, Rockville, MD, U.S.A.) were grown in DMEM/F12 (1:1 mixture of Dulbecco’s MEM and Ham’s F12; PAN Biotech, Aidenbach, Germany) supplemented with 10% fetal calf serum (Biochrom, Berlin, Germany) and were used at passages 25 to 30. Cells were counted with a Casy cell counter (Schärfe System, Reutlingen, Germany) before seeding. Viability of cultured cells was assessed microscopically by trypan blue exclusion test and flow-cytometrically by propidium iodide exclusion test. Unless otherwise described, in all experiments ARPE-19 cells were plated at µ-dish (35 mm, low, Ibidi, München, Germany) with a density of 1×10^4^ cells/cm^2^ and cultured for 24 or 48 hours at 37°C.

To inhibit the *de novo* formation of tunnelling nanotubes, latrunculin B (Invitrogen, Carlsbad, CA, USA) at a final concentration of 5 µM was added to the medium. Pluronic F-127 (Sigma-Aldrich Co., St. Louis, Missouri, USA) and DMSO (Sigma-Aldrich) was used as a solvent. To investigate microtubules in TNTs, the microtubule-destabilizing drug nocodazole (Sigma-Aldrich) was added to the medium at a final concentration of 15 µM.

### Microscopy and image analysis

For live time-lapse imaging, ARPE-19 cells were monitored with an Olympus IX81 inverted microscope equipped with Differential Interference Contrast (DIC) components and an integrated vital microscopy chamber (Olympus, Hamburg, Germany). Cells were imaged every 4 min at 37°C and 5% CO_2_. Image analysis was performed using celîR imaging software (Olympus). For long term experiments we used an automation to create movie-sequences.

For scanning electron microscopy (SEM), cells were fixed with 2.5% glutaraldehyde in 0.1 M cacodylate buffer and dehydrated using ethanol. After drying to the critical point, cells were sputtered with gold. A scanning electron microscope (model S430, LEO, Oberkochen, Germany) was used for cell analysis. All buffers, fixatives and embedding materials for electron microscopy were purchased from Serva (Serva, Heidelberg, Germany).

To stain mitochondria, cells were labelled with 1 µM JC-1 (Invitrogen) for 15 min at 37°C and visualized with an Olympus IX81 microscope. JC-1 selectively accumulates within the mitochondrial matrix and forms red fluorescent J-aggregates (emission 590 nm) in the presence of a highly negative transmembrane potential, but exists as green monomers (emission 530 nm) under depolarised conditions. The green phase was recorded using an excitation wavelength of 485 nm and an emission filter of 540/50 nm. The red phase of JC-1 was recorded using an excitation wavelength of 535 nm and an emission filter of 610/75 nm.

### Immunofluorescence

For immunohistochemistry of F-actin and tubulin cells were washed with PBS (PAA Laboratories GmbH, Austria), immediately fixed with 0.1% glutaraldehyde/4% paraformaldehyde (PFA) in PBS for 1 min followed by further fixation with 4% PFA for 15 min at room temperature (RT) as described in Önfelt et al. [21], [27], [30]. Unspecific antibody binding was blocked by incubating samples with 1% bovine serum albumin (BSA, Serva) for 30 min at RT. To visualize the actin cytoskeleton, cells were incubated with TRITC-conjugated phalloidin (Sigma-Aldrich) for 1 hour at 37°C. Nuclei were stained with DAPI (1 µg/ml, Sigma-Aldrich) for 5 min at RT. Indirect immunofluorescence labelling of tubulin was performed by using mouse anti-β-tubulin (1:30; Zymed, San Francisco, CA, USA) over night at 4°C. The secondary antibody FITC-conjugated rabbit anti-mouse antibody (1:100; Dianova, Hamburg, Germany) was used for 1 hour at RT. Samples were stored in 2% Dabco-glycerin/PBS as mounting solution at 4°C.

For immunostaining of Cx43 ARPE-19 cells were plated on 0.1 mg/ml poly-L-lysine-coated (PLL, Sigma-Aldrich) Met-Tek glass bottom culture dishes (MatTek Corporation, Ashland, MA, USA) at low density and cultured for 24 hours. Then cells were fixed in 2% formaldehyde with 0.2 M sucrose, 0.1 M PBS (pH 7.2) at RT for 20 min followed by permeabilization in 0.2% Triton X-100 in PBS at 4°C for 1 min. After blocking with 10% fetal calf serum in PBS for 20 min, cells were incubated for 1 hour with rabbit polyclonal anti-Cx43 antibody (1:250, Sigma-Aldrich) followed by incubation for 1 hour with 1:500 Alexa Fluor 647 goat anti-rabbit secondary antibody (Invitrogen) and Alexa Fluor 488 wheat germ agglutinin (WGA, Invitrogen) at RT. Imaging was performed on a Leica TCS SP5 confocal microscope (Leica Microsystems GmbH) with a 40×/1.25 NA oil-immersion objective.

### Monitoring intracellular Ca^2+^ by calcium imaging

To record intracellular Ca^2+^ level, 3 µM Fura-2 AM (Invitrogen), a Ca^2+^-specific vital dye, was used in staining solution containing 1.8 mg/ml Pluronic F127 and 0.3% BSA (Serva) in HBSS. First, cells were seeded on 2 cm ibidi culture dish with a concentration of 1×10^4^ cells/cm^2^ and incubated for 24 hours. After washing with HBSS, cells were incubated with the staining solution for 5 min. Then cells were washed with HBSS and incubated for another 10 min in 5% BSA in HBSS. Samples were washed and imaged with fluorescence microscopy.

For mechanical stimulation of single cells a micromanipulator (Leica Microsystems GmbH, Wetzlar, Germany) was used. The micropipette was back-loaded with sufficient volume of staining solution and closed on the end of the capillary tube. Two via TNT connected cells were chosen and one was carefully touched with the tip of the micropipette. Data was recorded and analyzed using the CelîR imaging software.

### Microinjection and micromanipulation with Lucifer Yellow and Dextran Red

To show the intercellular transfer of molecules we injected fluorescent dyes into cells: Lucifer Yellow (Invitrogen) for small molecules and Dextran Red (Invitrogen) for molecules sized 10,000 KDa. For microinjection, a FemtoJet/InjectMan NI 2 system (Eppendorf AG, Hamburg, Germany) was used with a Zeiss Axiovert microscope (Zeiss, Oberkochen, Germany). The micropipette was back-loaded with sufficient volume of the injection medium, containing Lucifer yellow with a concentration of 50 mM and Dextran Red 10,000 MW with 675 mM in water. The micropipette was then placed in the medium and the pressure in the micropipette was increased to 5 – 10 mbar. The loaded micropipette tip was brought into the field of view with a motorized, microprocessor-controlled micromanipulator. Microinjection was performed automatically with an angle of 45°. The pressure was increased up to 70 mbar.

### Exchange of organelles between ARPE-19 cells

For the description of organelle exchange two populations of ARPE-19 cells were cocultured for 24 hours according to Gurke et al. [32]. As organelle donor population, cells were labelled with membrane specific dye Vybrant DiD (Invitrogen), leading to red fluorescently labelled endocytic organelles. For the staining subconfluent cells were detached and suspended in 1 ml PBS with 4 µl DiD. Cells were incubated for 20 min and washed three times with fresh medium. Plated cells were incubated over night and medium were changed. As acceptor population, cells were labelled with cytoplasmic dye CellTracker Green (CTG, Invitrogen), which is not transferable between the cells. For the staining, subconfluent cells were washed twice with PBS and incubated in medium containing 1.5 mM CTG for 30 min. Then cells were incubated in fresh medium for another 30 min. DiD, CTG and not labelled cells were washed again with PBS, detached and suspended in fresh medium. For the experiment, CTG-labelled cells, DiD-labelled cells, mixed cells (CTG/DiD, 1:1) and controls were seeded. After 1 hour when the cells were attached to the culture dish, the medium was exchanged by fresh medium. Cells were analysed by flow cytometry and inspected with DIC- and fluorescence microscopy after 1 hour and 24 hours.

### Mechanical stimulations and membrane potential measurements

The measurement of TNT-dependent electrical coupling of ARPE-19 cells was preformed as previously described [25]. Briefly, cells were pre-loaded with 2 µM of DiBAC_4_(3) (Sigma-Aldrich) at 37°C for 45 min and exchanged with warm DMEM. Mechanical stimulations were applied by microinjection using a FemtoJet/InjectMan NI 2 system (Eppendorf) equipped with an Olympus IX70 microscope (Olympus) at 37°C and appropriate CO_2_. The tip of capillary was rapidly penetrated through the cell membrane and a bolus of PBS solution containing 0.2 mg/ml Cascade Blue (Invitrogen) was injected. Time-lapse fluorescence images (16 bit) were acquired before and after mechanical stimulation with a 60×/1.40 NA oil-immersion objective, a Polychrome V monochromator (T.I.L.L. Photonics GmbH, Gräfelfing, Germany), and an Andor DU-885 camera controlled by IQ 7.0 software (Andor Technology, Belfast, Northern Ireland). Cascade Blue and DiBAC_4_(3) were excited at 400 nm and 488 nm, respectively. Cells show increased fluorescence of DiBAC_4_(3) when there is depolarisation. The level of depolarisation was illustrated by pseudo-coloured intensity images acquired with ImageJ software (NIH, MA, USA). The quantification of fluorescence intensity of DiBAC_4_(3) was following the method as previously described [25].

## Supporting Information

Movie S1 **Time lapse video of migrating ARPE-19 cells and the formation of a nanotube.** The TNT freely moves in the medium and follows the migrating cells without making contact to the surface of the culture dish. The images were taken every 5 minutes for 3.3 hours using DIC microscopy. Scale bar, 50 µm.
(MP4)

Movie S2 **Time lapse video show calcium fluxes between two cells connected via a TNT (** **Fig. 4** **).** The Ca^2+^ level increase in the lower cell directly after manipulation and after 20 sec in the upper cell (red colour). Both cells recover close to normal level of Ca^2+^ after 45 sec. Scale bar, 20 µm.
(MP4)
